# Fiducial-based image-guided SBRT for pancreatic adenocarcinoma: Does inter-and intra-fraction treatment variation warrant adaptive therapy?

**DOI:** 10.1186/s13014-021-01782-w

**Published:** 2021-03-19

**Authors:** Colin S. Hill, Sarah Han-Oh, Zhi Cheng, Ken Kang-Hsin Wang, Jeffrey J. Meyer, Joseph M. Herman, Amol K. Narang

**Affiliations:** 1 Department of Radiation Oncology and Molecular Radiation Sciences, Johns Hopkins University School of Medicine, 401 N. Broadway, Suite 1440, Baltimore, MD 21231 USA; 2 Radiation Medicine, Zucker School of Medicine At Hofstra/Northwell, Lake Success, USA

**Keywords:** SBRT, Pancreatic adenocarcinoma, Active breathing coordination, Motion management, PTV design

## Abstract

**Purpose:**

Variation in target positioning represents a challenge to set-up reproducibility and reliability of dose delivery with stereotactic body radiation therapy (SBRT) for pancreatic adenocarcinoma (PDAC). While on-board imaging for fiducial matching allows for daily shifts to optimize target positioning, the magnitude of the shift as a result of inter- and intra-fraction variation may directly impact target coverage and dose to organs-at-risk. Herein, we characterize the variation patterns for PDAC patients treated at a high-volume institution with SBRT.

**Methods:**

We reviewed 30 consecutive patients who received SBRT using active breathing coordination (ABC). Patients were aligned to bone and then subsequently shifted to fiducials. Inter-fraction and intra-fraction scans were reviewed to quantify the mean and maximum shift along each axis, and the shift magnitude. A linear regression model was conducted to investigate the relationship between the inter- and intra-fraction shifts.

**Results:**

The mean inter-fraction shift in the LR, AP, and SI axes was 3.1 ± 1.8 mm, 2.9 ± 1.7 mm, and 3.5 ± 2.2 mm, respectively, and the mean vector shift was 6.4 ± 2.3 mm. The mean intra-fraction shift in the LR, AP, and SI directions were 2.0 ± 0.9 mm, 2.0 ± 1.3 mm, and 2.3 ± 1.4 mm, respectively, and the mean vector shift was 4.3 ± 1.8 mm. A linear regression model showed a significant relationship between the inter- and intra-fraction shift in the AP and SI axis and the shift magnitude.

**Conclusions:**

Clinically significant inter- and intra-fraction variation occurs during treatment of PDAC with SBRT even with a comprehensive motion management strategy that utilizes ABC. Future studies to investigate how these variations could lead to variation in the dose to the target and OAR should be investigated. Strategies to mitigate the dosimetric impact, including real time imaging and adaptive therapy, in select cases should be considered.

**Supplementary Information:**

The online version contains supplementary material available at 10.1186/s13014-021-01782-w.

## Introduction

Pancreatic adenocarcinoma (PDAC) carries a grim prognosis and is estimated to become the second leading cause of cancer death by 2030 [1]. Only half of patients present without clinical evidence of metastatic disease, and only a minority of patients without metastatic disease are amenable to upfront pancreatectomy at the time of diagnosis due to vascular involvement. Despite the high propensity for distant spread, local progression contributes significantly to the morbidity of the disease. A series of autopsies demonstrated that locally destructive growth may contribute to mortality in a third of patients [2]. Local progression may also significantly contribute to hospitalizations and in-hospital mortality, further highlighting the potential importance of local control [3]. With the availability of better systemic therapy regimens, the role of local control has become even more critical [4]. As such, radiation therapy is playing a larger role in the neoadjuvant and definitive setting [5–11]. In the neoadjuvant setting, radiation may improve outcomes with exploration in the setting of vascular involvement, both with respect to margin sterilization and local recurrence risk reduction [11–13]. In the definitive setting, radiation can add to local progression-free survival and prevent local obstructive complications as referenced above.

At our institution, it has been our practice to treat patients with borderline resectable or locally advanced pancreatic cancer with multi-agent chemotherapy for at least 4 months followed by SBRT to 33 Gy (Gy) in 5 fractions, either in the pre-operative or definitive setting [14, 15]. However, the proximity of radio-sensitive organs, particularly stomach and small and large bowel, makes delivering higher doses per fraction with SBRT challenging. This challenge is compounded further by the fact that the pancreas is a highly mobile organ, susceptible to positional variability with respiratory motion and bowel gas patterns, presenting a significant challenge to accurate targeting with highly conformal treatment [16]. Such variability can be assessed with on-board image guidance, most commonly cone beam computed tomography (CBCT). Due to the limited soft tissue contrast with CBCT, our institution utilizes endoscopically placed fiducials as a surrogate for tumor positioning, which allows for daily shifts off bony anatomy to the fiducials to account for inter-fraction positional variation. Importantly, reports on stereotactic therapy with on-board adaptive planning inform us that daily variation in anatomy can significantly impact the actual dose delivered to the tumor and organs at risk (OAR) [17]. As most sites do not have adaptive therapy capabilities, a better understanding of the magnitude of inter-fraction variation in this clinical setting can help inform appropriate margin design and treatment planning strategies. In addition to appreciating inter-fraction variation, an understanding of intra-fractionation variation in the positioning of these structures is also critical, as both have key implications with respect to margin design, pre-treatment image guidance, and intra-fraction monitoring strategies. Herein, we aim to characterize inter-fraction and intra-fraction variation in tumor positioning in patients treated with stereotactic body radiation therapy (SBRT) for PDAC at a high-volume institution.

## Methods

### Patient selection and treatment course

We reviewed 30 consecutive patients with borderline resectable or locally advanced PDAC who underwent 5-fraction SBRT to 33 Gy with volumetric-modulated arc therapy (VMAT) using alpha-cradle (Smithers Medical Products Inc., North Canton, OH, USA) or Vak-lok (CIVCO Medical Solutions, Coralville, IA, USA) immobilization with a wingboard (CIVCO Medical Solutions, Coralville, IA, USA); active breathing coordination (ABC, Elekta, Stockholm, Sweden); hexapod for rotational shifts; and daily image-guidance (IG) with CBCT. Prior to simulation, all patients underwent endoscopic fiducial placement with a goal of implanting three fiducials into the pancreas. For simulation and each fraction of the treatment course, patients were treated with an empty stomach after being nil per os (NPO) for at least 2–3 h. At simulation, patients were instructed to take a deep inspiratory breath hold (DIBH) preferably for 25 s with ABC technique, and an intravenous contrast-enhanced planning CT was acquired with the patient in DIBH, with a plan for treatment administration in DIBH.

Target volumes incorporated gross disease with coverage of involved vascular structures. Since 2017, the high-risk nodal basins around the celiac and superior mesenteric arteries have been included in the volume depending on the overall size of the target volume. Fiducials were contoured in the bone window for the purpose of fiducial matching at the time of treatment. VMAT plans were created for each patient. At each treatment fraction, CBCTs were performed to verify patient positioning prior to the start of treatment, whereby patients were aligned first to their bony anatomy and then shifted to the fiducials. Radiation was delivered during the duration of the breath-hold. During treatment, intra-fraction CBCT(s) were also acquired to confirm target positioning remained consistent throughout treatment, and additional shifts were made to re-align fiducials if needed.

### Quantification of the shift and statistical analysis

The last CBCT taken prior to treatment initiation for each fraction was selected to characterize the pre-treatment shift off of spine that was required to align fiducials, a measure of inter-fraction variation in tumor positioning. Similarly, the last intra-fraction CBCT acquired during each fraction was used to characterize intra-fraction variability in tumor positioning. As such, a total of ten CBCTs were reviewed for each patient’s treatment course, five pre-treatment and five intra-fraction CBCTs, resulting in a total of 300 CBCTs across 30 patients. Utilizing Velocity software (Varian Medical Systems, Inc., Palo Alto, CA, USA) the selected CBCTs for each fraction were fused to the simulation CT using the bony anatomy of the spine from the simulation as the reference point. The CBCT was then shifted to align the fiducials from the inter-fraction CBCT with the position of the fiducials on the planning simulation scan, and the shift in the superior-inferior (SI), left–right (LR), and anterior–posterior (AP) axes was then quantified. To quantify the intra-fraction shift, the shift from bone to the fiducials using the intra-fraction CBCT was subtracted from the inter-fraction shift from bone to the fiducials. The magnitude of the shift was also calculated as a three-dimensional (3D) vector. The mean, maximum, and standard deviation (SD) of the shift in each axis, including the 3D vector, were calculated for 30 individual patients over 5 fractions. In order to provide recommendations for margin design, with the assumption that pre-treatment imaging would account for inter-fraction variation and planning tumor volume (PTV) margin would primarily account for intra-fraction margin, systematic and random components of intra-fraction variation were calculated and combined to form a recommended PTV margin, as has been previously described [18]. Additionally, we also wanted to explore if there was an association between inter-fraction and intra-fraction variation, with the application being that if large inter-fraction variation is appreciated early in the treatment course, this may serve as a predictor of intra-fraction variation as well, which could help inform intra-fraction monitoring strategies and reconsideration of margin design. To explore this relationship, a linear regression analysis with the Pearson correlation test was conducted to investigate the relationship between the pre-treatment shift and the intra-fraction shift.

## Results

### Characterizing inter-fraction variation

**Fig. 1:**
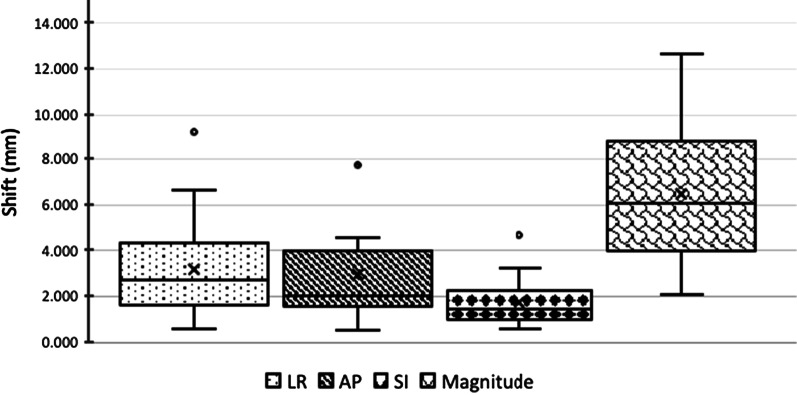
Inter-fraction shift variation

Across 150 pre-treatment CBCTs (five fractions for 30 patients), the mean pre-treatment shift from spine to fiducial in the LR, AP, and SI axes was 3.1 ± 1.8 mm, 2.9 ± 1.7 mm, and 3.5 ± 2.2 mm, respectively, and the average magnitude of the vector shift was 6.4 ± 2.3 mm. For each patient, maximum shifts were also characterized across the five fractions of treatment. On average, the maximum pre-treatment shift in the LR, AP, and SI axes was 5.5 ± 2.9 mm, 5.1 ± 2.8 mm, and 6.6 ± 3.9 mm, respectively, and the average maximum of the magnitude of the vector shift was 9.3 ± 4.2 mm. Figure 1 presents a summary of the mean pre-treatment shift variation across all three axes and the along the vector.

### Characterizing the intra-fraction variation

**Fig. 2:**
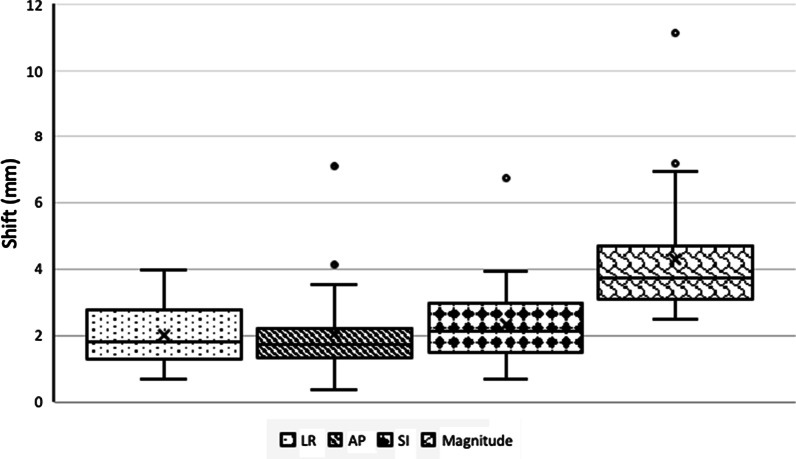
Intra-fraction shift variation

Mean intra-fraction shifts were characterized across 150 intra-fraction CBCTs. On average, the mean intra-fraction shift in the LR, AP, and SI directions were 2.0 ± 1.0 mm, 2.0 ± 1.3 mm, and 2.3 ± 1.2 mm, respectively, and the mean intra-fraction vector shift was 4.3 ± 1.8 mm. On average, the maximum of the intra-fraction shift in the LR, AP, and SI directions were 4.3 ± 2.1 mm, 4.1 ± 2.8 mm, and 5.3 ± 3.5 mm, and the maximum of the intra-fraction vector shift was 7.3 ± 3.8 mm. Figure 2 presents a pictorial summary of the intra-fraction shift variation across all three axes and the along the vector. Utilizing von Herk calculations, combining systematic and random components of intra-fraction margin results in PTV margin recommendations of 3.7 mm in the LR axis, 4.4 mm in the AP axis, and 4.8 mm in the SI axis [19].

### Linear regression relationship of inter- and intra-fraction variation

A linear regression model was conducted to investigate the relationship in the absolute mean amount of the inter-fraction shift to the absolute mean intra-fraction shift (Table 1, Additional file 1: Fig. S1A–S1D). Along the LR axis, there was no significant relationship between the inter-fraction and the intra-fraction shifts. The shift along the SI and AP axes and the magnitude of the vector shift showed a significant relationship between the inter-fraction and intra-fraction shifts in the model. The shift along the AP axis and for the vector showed moderate correlation between the inter- and intra-fraction shifts (r = 0.57 and r = 0.55, respectively). The shifts in the SI axis only showed weak correlation (r = 0.40). For example, using the model, a 5 mm increment in the mean inter-fraction shift along the SI axis would be associated with a 1.8 mm intra-fraction shift.

**Table 1:** Linear regression relationship between inter- and intra-fraction variation

*LR axis* Mean abs. Intra-fraction and mean abs. Inter-fraction shift (*p* = 0.1228, r = 0.288)
*SI axis* Mean abs. Intra-fraction = 0.13 × (mean abs. Inter-fraction shift) + 1.18 (*p* = 0.0262, r = 0.41)
*AP axis* Mean abs. Intra-fraction = 0.28 × (mean abs. Inter-fraction shift) + 1.17 (*p* = 0.001, r = 0.57)
*Vector* Mean abs. Intra-fraction = 0.25 × (mean abs. Inter-fraction shift) + 2.27 (*p* = 0.018, r = 0.55)

Abs. = absolute

## Discussion

Even with a comprehensive motion management strategy with respiratory gating and a breath-hold technique, both inter-fraction and intra-fraction variation remain significant for patients undergoing SBRT for pancreatic cancer. Moreover, for both inter-fraction and intra-fraction variation, the maximum shift, on average, was approximately twice the mean shift, highlighting the wide range of potential variability in tumor positioning that occurs over the course of a treatment course. The dosimetric impact of such variation in the applied shifts are not well understood and may have significant implications for treatment failure and toxicity. Strategies therefore to account for such variation are critical when treating this patient population, as is a better understanding of sources of variation.

To our knowledge, this is the largest series to date in the literature that has characterized inter-fraction and intra-fraction variation in tumor positioning for patients undergoing SBRT for pancreatic cancer with the breath-hold technique. Yang et al. reported on eleven patients but only seven patients had pancreatic tumors, and a free-breathing technique was used [20]. Another series reported on five patients treated with intensity modulated radiation for pancreatic cancer and respiratory gating with treatment during the expiration phase [21]. The mean shift off bony anatomy to the fiducial was 1.8, 1.6, and 4.1 mm in the LR, AP, and SI directions, respectively, with the SI shift being comparable to our finding (3.5 mm), but two patients were post-resection so this cohort is not fully representative of patients receiving radiation to an intact pancreas [21]. A comparable study to ours reported on 19 pancreatic cancer patients treated with SBRT using the breath-hold technique, with the mean inter-fraction shift off bone to fiducial of 1.5, 2.0, and 3.0 mm in the LR, AP, and SI axes, respectively [22]. Although treatment verification involved a CBCT and 2-dimensional (2-D) kilo-voltage (kV)-projection images prior to beam delivery on day 1, the subsequent shifts for fractions 2–5 were measured using breath-hold kV-projection images with the day 1 kV-projections serving as the reference point rather than the planning CT scan [22]. Regarding intra-fraction variation, only a few studies to date that have characterized this for pancreatic tumors with DIBH. Acknowledging its limitations, the aforementioned study reported that the intra-fraction variation with DIBH was within the 2 mm PTV margin volume [22]. Utilizing end-exhalation breath-holds, Nakamura et al. reported intra-fraction variation along the LR, AP, and SI axes, respectively of 0.0 ± 1.1 mm, 0.1 ± 1.2 mm, and 0.1 ± 1.0 mm [23]. However, end-exhalation is difficult for patients to achieve compared to a DIBH. Mean intra-fractional variation utilizing real-time tumor tracking report comparable numbers to our study: 0.8 mm, 0.6 mm, and 1.7 mm in the LR, AP, and SI directions, respectively [24].

Inter-fraction variation is assumed to be a combination of several factors such as daily variation in the bowel gas patterns, variation in respiratory-induced movement of the pancreas, and clinical set-up variability. Consistent with our findings, the largest degree of movement has been reported to be in the S-I direction [24–30]. Strategies to address these factors should be considered. With respect to respiratory-induced motion, it is unclear whether there are differences in contribution to inter-fractional variation between various motion management strategies, including ABC, gating, abdominal compression, or a free-breathing approach. While our institution preferentially favors the ABC technique, this technique is challenging for patients to master, as it requires them to follow a complex series of synchronized instructions, which are often introduced in a compressed timeframe during simulation. The complexity of the ABC technique can create anxiety, which may contribute to variation in respiratory motion. Earlier introduction of the ABC technique along with thorough patient education and coaching are important, as may be the development of systems that give real-time visual feedback of the respiratory trace to the patient during treatment. With respect to bowel gas patterns, we now employ a strategy of having patients NPO for five hours prior to treatment to ensure gastric emptying. How to address variation in small and large bowel filling is less clear. Whether scheduled use of simethicone or specific dietary recommendations prior to treatment would be helpful is uncertain, but strict dietary control for this patient population is challenging, as they are often struggling with nutritional deficiency in the setting of pancreatic insufficiency and chemotherapy-related side effects.

While the lack of resolution on CBCT prevented precise characterization of the dosimetric impact of inter-fraction variation, the experience with magnetic resonance-guided radiation therapy (MRgRT) has suggested that the dosimetric impact on both OARs *and* target coverage is not insignificant [17]. Notably, within a target volume, there may be varying degrees of inter-fraction variation, as tumor involvement of relatively rigid structures such as the vasculature may not necessarily exhibit the same degree of movement as the rest of the tumor, a concept that may be particularly true in the setting of elective coverage defined by the peri-pancreatic vasculature. Indeed, the MRgRT experience has demonstrated the high frequency with which adaptive planning is necessary due to inter-fraction variation. However, recent reports using MRgRT have demonstrated that, with the ability to employ adaptive radiotherapy (ART), safe administration of high biologically equivalent doses (BED) may be feasible, which is encouraging given data suggesting value in dose escalation for this disease. Indeed, some investigators have been able to safely administer treatment schedules with a BED of 100 Gy, which has produced encouraging local control outcomes in other disease sites [31, 32]. In addition to the value that ART may have in this setting, utilization of robust optimization algorithms that account for potential inter-fraction variation, which has primarily been used in the particle therapy space, may also be a consideration [33, 34]. Planning strategies that help screen for patients exhibiting greater degrees of variation will be useful to determine which patients need adaptive therapy or re-planning since this is a resource intensive process [35].

Those factors that contribute to inter-fraction variation can similarly contribute to intra-fraction variation. We postulate that variation in respiratory patterns may be the largest contributor to intra-fraction variation and may benefit from the strategies referenced above. Whether bowel gas patterns change significantly during the half hour over which most patients completely treatment is unclear and should be characterized. What is clear is the fact that the magnitude of the intra-fraction variation is not insignificant. Therefore, frequent on-board imaging or continuous monitoring, such as real-time tumor tracking, should be considered to minimize the risk of under-dosing target volumes and delivering higher than desired doses to OARs. For patients demonstrating large inter-fraction variation, our correlation studies emphasize the importance of frequent monitoring for these patients as there may be a correlation with greater degrees of intra-fraction variation. Intra-fraction magnitude should also inform appropriate design for target volumes. We suggest taking multiple ABC scans at simulation, at least three, and then applying an asymmetric expansion for the internal tumor volume to account for the intra-fractional variability with the breath-hold at treatment. As previous studies have shown, a comprehensive understanding of tumor motion can inform margin design [36–38]. On a population level, our analysis suggests that to achieve a 95% likelihood of tumor coverage while accounting for random and systematic error with intra-fraction variability, the radial for the internal volume with SBRT for pancreatic tumors should be approximately 4.0–4.5 mm and the SI margin should be approximately 4.5–5.0 mm [36]. Re-planning should be considered if patients exhibit greater variation with fiducial matching during treatment to minimize the dosimetric impact of this variation, especially with respect to potential toxicity to surrounding organs at risk.

The limitations in our series are inherent to retrospective analysis from a single institution. Although our series is large compared to similar reports in the literature, we are still limited by a small patient cohort. In addition, patients were treated after being NPO only for 2–3 h and this may have introduced more variability as our practice is now to treat after being NPO for about 5 h. Although we did not appreciate any significant fiducial migration during treatment for our patient cohort, we cannot rule out the possibility of sub-clinical migration. Furthermore, intra-fraction CBCT assessment was acquired only once per fraction and may not fully encapsulate the full range of transitory changes that occurring during treatment. Additionally, when significant, correlation values between inter- and intra-fraction shifts were only weak or moderate.

## Conclusion

Clinically significant inter- and intra-fraction variation occur during treatment of pancreatic cancer with SBRT even with a comprehensive motion management strategy that utilizes the breath-hold technique. Further characterization across motion management strategies should be pursued, as should strategies to mitigate that size and dosimetric impact of this variation.

## Supplementary Information

**Additional file 1.** Linear regression relationship between Inter- and Intra-fraction variation in the left–right axis (1A), anterior–posterior axis (1B), superior–inferior axis (1C), and the vector (1D).

## Data Availability

All data generated or analyzed during this study are included in the published article (and its supplementary information files).

## Abbreviations

SBRT: Stereotactic body radiation therapy
PDAC: Pancreatic adenocarcinoma
OAR: Organs-at-risk
ABC: Active breathing coordination
SI: Superior–inferior
LR: Left–right
AP: Anterior–posterior
Gy: Gray
CBCT: Cone beam computed tomography
VMAT: Volumetric-modulated arc therapy
IG: Image-guidance
NPO: Nil per OS
DIBH: Deep inspiratory breath hold
3D: Three-dimensional
SD: Standard deviation
PTV: Planning tumor volume
2-D: 2-Dimensional
kV: Kilo-voltage
MRGRT: Magnetic resonance-guided radiation therapy
BED: Biologically equivalent dose
ART: Adaptive radiotherapy
