# Supplementary material for: Fiducial-based image-guided SBRT for pancreatic adenocarcinoma: Does inter-and intra-fraction treatment variation warrant adaptive therapy?
Source: Radiat Oncol. 2021 Mar 19;16:53. doi: 10.1186/s13014-021-01782-w (PMC7980583; doi:10.1186/s13014-021-01782-w)

**Supplementary Figures 1A-1D:** Linear Regression relationship between Inter- and Intra-Fraction Variation in the Left-Right axis (1A), Anterior-Posterior axis (1B), Superior-Inferior axis (1C), and the Vector (1D).

**Figure 1A**
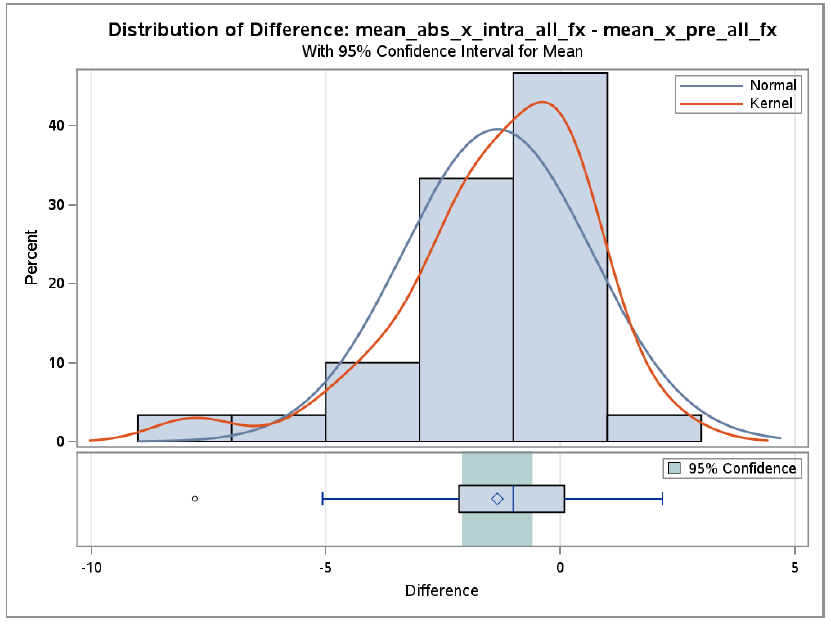


**Figure 1B:**
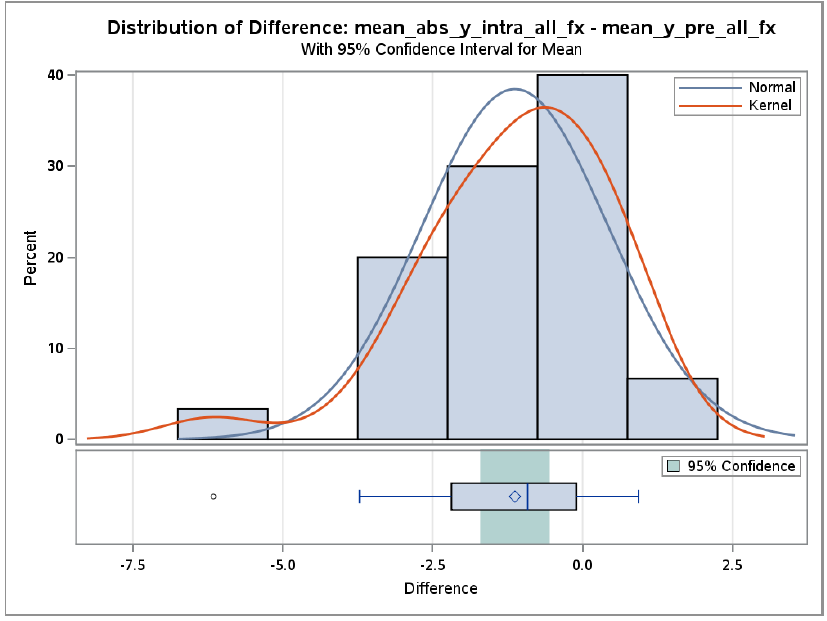


**Figure 1C:**
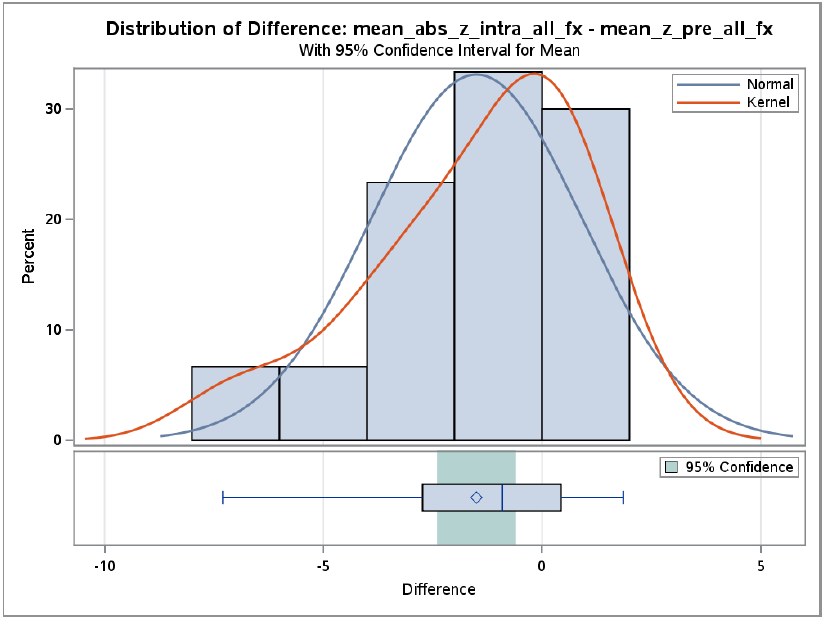


**Figure 1D:**
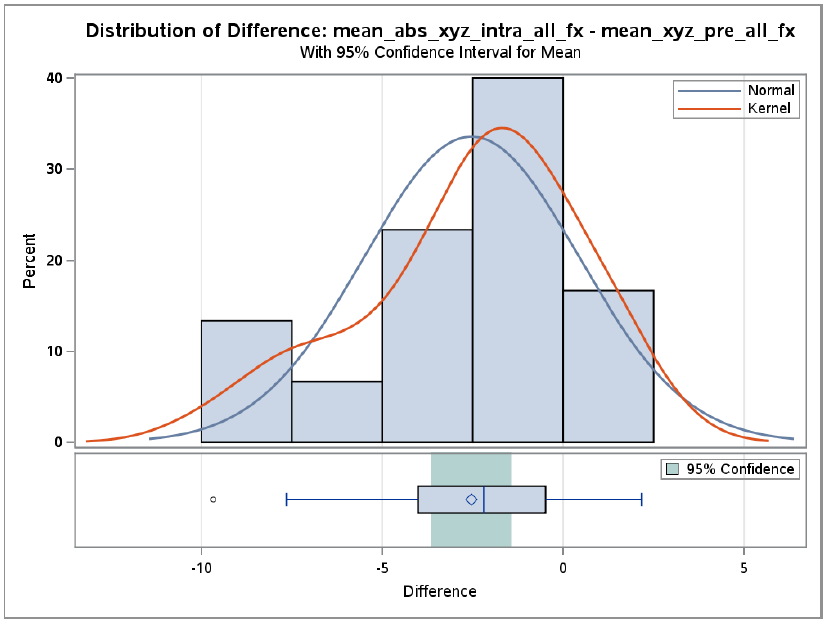

Supplement: Supplementary file 1 — Additional file 1. Linear regression relationship between Inter- and Intra-fraction variation in the left–right axis (1A), anterior–posterior axis (1B), superior–inferior axis (1C), and the vector (1D). [file 13014_2021_1782_MOESM1_ESM.docx]
